# Randomized trial of the efficacy and safety of a new oral spray for drug‐induced xerostomia

**DOI:** 10.1002/cre2.29

**Published:** 2016-05-12

**Authors:** Frank Donath, Françoise Tonner, Rajeev Chavda, Jean‐Philippe Gatignol, Julie Bouyrie

**Affiliations:** ^1^ SocraTec R&D GmbH Erfurt Germany; ^2^ Institut Recherche Pierre Fabre Toulouse France; ^3^ Medical Department Pierre Fabre Oral Care Castres France; ^4^ Pierre Fabre Medical Devices Toulouse France

**Keywords:** Efficacy, oral spray, safety, tolerability, xerostomia

## Abstract

The aim of this study was to evaluate the efficacy, safety, and tolerability of three formulations of DC161 oral spray, a saliva substitute, and a comparator in relieving drug‐induced xerostomia. This was an open‐label, randomized, 4‐period, cross‐over study in adult subjects with drug‐induced xerostomia and documented hyposalivation. During each of the four 1‐day periods, one product (one of three DC161 formulations or the comparator) was applied at T0 and then at T4h (before a meal). Mouth dryness and related symptoms were evaluated by the subject on a 100‐mm visual analog scale. The primary efficacy criterion was the area under the curve of the dry mouth evaluation (baseline to T4h) after the first application. The oral mucosa was examined by a dental specialist; tolerability and product acceptability were assessed by the subject. Twenty‐four subjects were randomized and completed the study. Despite large variability in data among the products, the selected aqueous formulation – DC161‐DP0292 – reduced the intensity of dryness of mouth at least as well as the comparator; DC161‐DP0292 provided a fast relief and a long‐lasting effect on mouth dryness. Both products improved other symptoms such as swallowing and speaking, even when applied just prior to a meal. DC161‐DP0292 was well tolerated and rated by subjects as providing a slightly higher acceptability of taste/aftertaste, texture, and lubricating effect than the comparator. No clinically relevant signs were reported for any product following the oral examination. DC161‐DP0292 provides fast and long‐acting symptomatic relief and is a relevant new treatment for drug‐induced xerostomia.

## Introduction

Dry mouth or xerostomia, a symptom of salivary hypofunction, is a common side effect of many medications and is also associated with various medical conditions. As the number of prescribed drugs increases with age, drug‐induced salivary hypofunction and dry mouth complaints become an increasing health problem. More than 500 commonly used medications can cause xerostomia; these include antidepressants, antihypertensives, opiates, bronchodilators, proton‐pump inhibitors, antipsychotics, antihistamines, diuretics, antineoplastics, and others (Gupta *et al*., 2006; Mouly *et al*., 2007).

Estimates of the prevalence of persistent dry mouth vary between 10% and 50%. Prevalence is conservatively estimated as approximately 20% in the general population, with increased prevalence in women (up to 30%) and in the elderly (up to 50%) (Orellana *et al*., 2006; Hopcraft and Tan, 2010; Villa and Abati, 2011). Dry mouth is often attributed to increased prevalence of chronic illnesses that require pharmacological treatments with dry mouth as a side effect (Porter *et al*., 2004; Thelin *et al*., 2008). Although salivary flow does not necessarily decrease with age, the synergistic effects of combining medications also contribute to xerostomia, and therefore, the elderly are more likely to suffer from dry mouth. Thus, while drug‐induced xerostomia is generally reversible, the disorders for which these medications are prescribed are frequently chronic.

A wide range of therapies are available for the management of xerostomia, including sialagogues and saliva substitutes, as well as general measures like sipping water or chewing gum. However, the effectiveness of many available agents is controversial, and few have been tested in controlled clinical trials. To address this concern, Pierre Fabre Medical Devices has developed three formulations (two aqueous and one oily) of a new oral spray, DC161, which acts as a saliva substitute for the treatment of xerostomia in adults. The aim of these formulations is to act as temporary substitutes for saliva by recreating its physical action without disturbing the oral environment. Their mechanisms of action vary according to the different physical and chemical properties of their components.

The purpose of the current study was to evaluate the clinical efficacy of these three DC161 formulations and an active comparator – Aequasyal® oral spray (Carilène Laboratory, Montesson, France), a marketed oily saliva substitute – allowing comparison of outcomes based on the product formulation. Relief of drug‐induced xerostomia and other associated symptoms and the effects on the oral mucosa were assessed, as well as local tolerability and product acceptability.

## Subjects and Methods

### Subjects and study design

This was an exploratory, randomized, open‐label, active‐controlled, 4‐period, cross‐over study conducted at a single center in Germany between 7 January 2014 and 21 February 2014. The study was approved by the Landesärztekammer Thüringen Ethics Committee on 28 November 2013, and performed in compliance with the Declaration of Helsinki, Good Clinical Practice Guidelines (CPMP/ICH/135/95), and the EN ISO 14155 (2011) standard for medical devices. All subjects provided written informed consent prior to study participation. The study (EUDAMED no. CIV‐13‐10‐011648) was registered at www.clinicaltrials.gov (NCT02005328).

Male and female subjects (18 years and older) were eligible if they had taken drug(s) causing salivary hypofunction/ xerostomia for at least 1 week prior to study initiation and were expected to continue without any change during the study. Most of these subjects were taking chronic medications like beta blockers, antacids, anticholinergics, and psychotropic drugs. Subjects were also required to have a score of ≥40 mm on a 100 mm visual analog scale (VAS) rating dryness of mouth and documented hyposalivation (resting saliva weight ≤0.5 g absorbed over 5 min using a swab method) (Navazesh and Christensen, 1982). Exclusion criteria included bucco‐dental disease, which may have interfered with study conduct; history of head and neck irradiation and cancer chemotherapy; Sjögren syndrome and related autoimmune diseases or other medical causes of xerostomia (oral candidiasis); or a history of major medical/psychiatric illness or surgery.

Eligible subjects were equally randomized to one of four treatment sequence groups (ADBC, BACD, CBDA, or DCAB; where A = Aequasyal®, B = DC161‐DP0291, C = DC161‐DP0292, and D = DC161‐DP0293) according to a computer‐generated randomization list provided by the *Institut de Recherche Pierre Fabre*. Subjects were enrolled by Dr Frank Donath at SocraTec R&D GmbH (Germany). The compositions of the four products evaluated in this study as well as a presentation of the differences in their mechanism of action according to the formulation are provided in Table 1.

**Table 1:** Composition of study products and mechanism of action.

	*Study product*			
	DC161‐DP0291	DC161‐DP0292	DC161‐DP0293	Aequasyal®^a^
Formulation	Aqueous solution	Aqueous solution	Oily solution	Oily solution
Mode of administration	Spray	Spray	Spray	Spray
Composition	Glycerol, *polysorbate 80*, *soja lecithin*, *sodium hyaluronate*, xanthan gum, potassium chloride, xylitol, anhydrous disodium hydrogen phosphate, potassium dihydrogen phosphate, *sucralose*, *soft mint flavor*, macrogolglycerol 40 hydroxystearate, benzylic alcohol, *sodium benzoate*, *cetylpyridinium chloride*, *alpha tocopheryle acetate*, and pure water.	Glycerol, *povidone K30*, *copovidone*, xanthan Gum, potassium chloride, xylitol, *marshmallow concentrated hydroglycerined extract*, anhydrous disodium hydrogen phosphate, potassium dihydrogen phosphate, macrogolglycerol 40 hydroxystearate, *potassium sorbate*, benzylic alcohol, and pure water.	Paraffin liquid, cotton refined oil, orange flavor, and alpha tocopheryle acetate.	94.4% triesters of glycerol oxidized fatty acids of vegetal origin (corn oil); silicium dioxide; food aromas: orange, grapefruit, and mint; and aspartame.
Mechanism of action	Moisturizing effect as an aqueous solution, which spreads over and is retained on the mucosal surface by its surfactant and humectant properties.	Moisturizing effect as an aqueous solution, which spreads over and is retained on the mucosal surface by its coating and thickening agents and its humectant effects.	Formation of a protective barrier against dryness by reducing moisture loss from tissue and provide a lubricating effect. Properties provide adherence to the oral mucosa, thus retaining the product in the oral cavity.	The effects are 3‐fold: lubrication; provision of adherence properties due to the formation of a lipid film, which reduces the loss of water and restores viscoelasticity of the oral mucosa; and protection against local infections.

Differences between the compositions of the two DC161 aqueous solutions are displayed in italics.

a Leaflet, July 2010 (translation from French)

During each of the four 1‐day study periods (P1, P2, P3, and P4), one of the four test products was applied twice a day, at T0 and at T4h, such that each subject received a total of eight applications of product during the study. The four periods were separated by a washout of 3 days maximum. All applications were performed by the subject at the study center under the investigator's supervision. The two applications of each product were separated by 4 h; the first application was performed after breakfast (the subject had fasted for at least 1 h before the first application), and the second application was performed 15 min before a standard meal. Fluid intake was standardized: for each period, no fluid was permitted from 30 min before until 2 h after the first application; from 2–6 h after the first product application, fluid intake was limited to two glasses of water (2×150 mL).

The three DC161 formulations were applied via three sprays in the mouth – one spray inside each cheek and one spray on the tongue. Application of the comparator was performed according to the instructions for use, that is, two sprays in the mouth, one spray inside each cheek, and distribution of the solution in the mouth with the tongue. As the application scheme differed between the comparator spray and the three DC161 sprays, the study was not blinded.

### Clinical assessments

#### Subject‐reported assessments

Dryness of mouth and other associated symptoms were evaluated using a 100 mm VAS (where 0 mm = “no symptoms” and 100 mm = “the worst imaginable symptoms”) for the following 8 items: 1 = difficulty in speaking; 2 = difficulty in swallowing; 3 = saliva in mouth; 4 = dryness of your mouth; 5 = dryness of throat; 6 = dryness of lips; 7 = dryness of tongue; and 8 = level of thirst. During each period, the assessment was self‐performed by the subject 5 min before the first product application (T0) and at 5, 10, 20, 30, and 40 min and 1, 1.5, 2, 3, and 4 h after the first product application; then at 30 min, 1, 1.5, and 2 h after the second application (i.e. T4.5 h, T5h, T5.5 h, and T6h).

Local tolerability was self‐assessed by the subject using a 4‐point ordinal scale (0 = none, 1 = mild, 2 = moderate, and 3 = severe) evaluating painful tongue, burning sensation, and tickling after the first and second applications, at T4h (before the second application) and at T6h.

Product acceptability was evaluated by the subject under investigator guidance using a 100 mm VAS for assessment of taste, ease of spread, lubrication, ease of use, and sensations in mouth (burning, tickling, and irritation), after the first and second applications (at T4h [before the second application] and at T6h).

#### Investigator‐reported assessments

Safety was assessed via evaluation of the mouth mucosa by the same dental specialist for all subjects. This assessment was carried out using a 4‐point ordinal scale (0 = none, 1 = mild, 2 = moderate, and 3 = severe) measuring redness, dryness of the tissues, and degree of inflammation before the first and second applications (T0 and T4h) and after the second application (at T6h).

A global physical examination was carried out by the investigator and was evaluated as normal or abnormal, with further investigation for any abnormal findings.

All subject‐assessed local reactions with a score >0 were reported by the investigator as adverse events, as were all other adverse events related to safety.

#### Outcomes

The primary efficacy outcome was the dry mouth evaluation (item 4 of VAS assessment) over 4 h after the first product application (T0–T4h). Secondary efficacy outcomes included the dry mouth evaluation (VAS item 4) over 2 h after the second application (T4h–T6h) and improvement of other dryness‐related symptoms (i.e. VAS items 1, 2, 3, 5, 6, 7, and 8) after the first and second applications (T0–T6h). Other secondary evaluation outcomes included the following: time to onset of action (defined as a reduction of at least 20% from baseline); time to maximum effect; and duration of action after the first and second applications, defined as the time interval between first and last time point where a 20% reduction in VAS from baseline was observed.

#### Statistical analysis

A sample size of 24 subjects was arbitrarily chosen. Results are regarded as exploratory in nature, and only descriptive statistics were performed.

Two analysis sets were defined. The full analysis set (FAS) included all randomized subjects who received each product of their sequence group and had data available for the primary efficacy criterion for each product. The safety set comprised all randomized subjects who received at least one product application of their sequence group.

The primary efficacy endpoint was the area under the curve (AUC) of the dry mouth evaluation (VAS item 4) from baseline to 4 h after the first product application. The primary analysis was performed on the FAS and was descriptive only. The adjusted AUC difference between groups and the associated 95% confidence interval (CI) were calculated using an analysis of variance, including product and period as fixed effects and subjects as random effect on the FAS. All secondary efficacy criteria were analyzed using the analysis of variance model described for the primary analysis, by product group on the FAS. Time to onset of action and time to maximum effect were described according to the Kaplan–Meier method.

The duration of action after the first and second applications was described by product using number of subjects, number of missing data, mean, 95% CI of the mean, standard deviation (SD), minimum, and maximum.

The safety set was used for all safety analyses. Any reported adverse event starting during a period *X* until the first application of the next period (*X* + 1) was attributed to the product applied in period *X*.

## Results

### Subjects and demographics

A total of 40 subjects were screened; of these, 16 did not meet the study eligibility criteria, and 24 subjects (17 women [70.8%] and 7 men [29.2%]) were randomized. All 24 subjects completed the study, and therefore, the FAS and safety set were equivalent.

Subject demographics and baseline characteristics are shown in Table 2. Overall, mean subject age was 66.8 years (SD: 9.1), and mean body mass index was 30.8 kg/m^2^ (SD: 4.8). At baseline, the mean VAS score for dryness of mouth (item 4) for all subjects was 73.8 mm (SD: 11.9). Mean saliva weight (collected over 5 min) was 0.31 g (SD: 0.13) overall, and values were similar for the four sequence groups. The most frequently reported concomitant diseases were hypertension (reported by 20 subjects [83.3%]) and Type 2 diabetes mellitus (6 subjects [25.0%]).

**Table 2:** Subject demographics and baseline characteristics.

		Treatment sequence				
		ADBC *n* = 6	BACD *n* = 6	CBDA *n* = 6	DCAB *n* = 6	Total *n* = 24
Age (years)	Mean (SD)	63.2 (9.5)	68.8 (5.8)	62.8 (12.3)	72.5 (5.2)	66.8 (9.1)
	Median (range)	66.5 (44–70)	69.0 (59–76)	66.5 (41–73)	72.5 (64–80)	69.0 (41–80)
Gender	Female	4 (66.7%)	3 (50.0%)	5 (83.3%)	5 (83.3%)	17 (70.8%)
	Male	2 (33.3%)	3 (50.0%)	1 (16.7%)	1 (16.7%)	7 (29.2%)
BMI (kg/m^2^)	Mean (SD)	29.010 (3.329)	31.789 (6.513)	32.914 (5.889)	29.296 (2.360)	30.752 (4.819)
	Median (range)	28.405 (25.08–34.45)	30.736 (25.78–43.14)	34.150 (25.86–40.98)	28.787 (27.17–33.78)	28.902 (25.08–43.14)
Salivation test at baseline	Mean (SD)	0.32 (0.15)	0.32 (0.12)	0.30 (0.17)	0.32 (0.13)	0.31 (0.13)
	Median (range)	0.25 (0.2–0.5)	0.30 (0.2–0.5)	0.25 (0.1–0.5)	0.30 (0.2–0.5)	0.30 (0.1–0.5)
Dry mouth evaluation at baseline (VAS item 4)	Mean (SD)	77.3 (9.4)	76.2 (7.8)	71.3 (11.5)	70.5 (18.0)	73.8 (11.9)
	Median (range)	81.5 (59–84)	79.0 (62–84)	75.5 (57–83)	66.5 (45–97)	77.5 (45–97)

A = Aequasyal®, B = DC161‐DP0291, C = DC161‐DP0292, D = DC161‐DP0293.

BMI, body mass index; SD, standard deviation; VAS, visual analog scale.

### Efficacy

#### First product application: T0–T4h

Results for the primary efficacy criterion are shown in Table 3 for the oral spray formulations DC161‐DP0291, DC161‐DP0292, DC161‐DP0293, and the comparator. Data were highly variable, as reflected by the SDs of the mean AUC for dryness of mouth (VAS item 4) as well as the standard errors (SEs) and 95% CIs of the differences between products: all CIs cross zero indicating a lack of statistical significance for all comparisons. However, of the three DC161 formulations tested, only DC161‐DP0292 showed a reduction in mouth dryness versus the comparator: The adjusted mean difference in AUC (T0–T4h) versus the comparator was −935.00 (SE: 577.70; 95% CI: −2088.42, 218.42) for DC161‐DP0292, compared with 350.27 (SE: 577.70; 95% CI: −803.15, 1503.69) for DC161‐DP0291, and 146.02 (SE: 577.70; 95% CI: −1007.40, 1299.44) for DC161‐DP0293.

**Table 3:** Primary efficacy analysis: AUC (T0–T4h) for dryness of mouth (item 4 of VAS) – ANOVA values (FAS).

Dryness of mouth		DC161‐DP0291 *n* = 24	DC161‐DP0292 *n* = 24	DC161‐DP0293 *n* = 24	Aequasyal® *n* = 24
AUC (T0–T4h) of VAS item 4	Mean (SD)	11326.52 (5407.47)	10041.25 (4525.68)	11122.27 (4648.37)	10976.25 (5140.21)
Adjusted AUC (T0–T4h) difference between product and comparator	LSM (SE)	350.27 (577.70)	−935.00 (577.70)	146.02 (577.70)	—
	[LSM 95% CI]	[−803.15; 1503.69]	[−2088.42; 218.42]	[−1007.40; 1299.44]	—

AUC, area under the curve; VAS, visual analog scale; ANOVA, analysis of variance; FAS, full analysis set; SD, standard deviation; LSM, least square mean; SE, standard error; CI, confidence interval.

Based on these results, DC161‐DP0292 (Elgydium Clinic Dry Mouth, Pierre Fabre Oral Care, France) was selected, and therefore, further results will focus on this formulation compared with the comparator.

**Figure 1:**
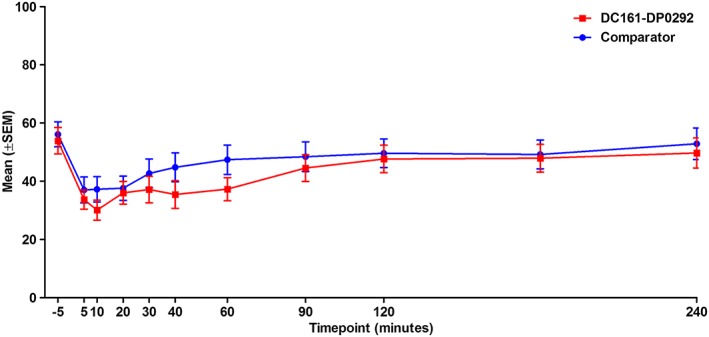
Values for visual analog scale item 4 (dryness of mouth) from T0 to T4h (expressed as geometric mean).

Figure 1 shows the mean VAS scores (mm) for dryness of mouth (item 4) for DC161‐DP0292 and the comparator after the first product application from T0 to T4h (240 min). Mean VAS scores at baseline (before T0) were comparable between products: 53.9 mm (SD: 22.3) for DC161‐DP0292 and 56.1 mm (SD: 21.1) for the comparator. For DC161‐DP0292, the mean change in VAS score for dryness of mouth was −20.2 mm (SD: 20.8) after 5 min, with a further decrease after 10 min (−23.8 mm [SD: 21.5]). For the comparator, the maximum effect was observed after 5 min (−19.1 mm [SD: 16.5]). By the end of the first dosing interval, the residual mean change from baseline was −4.2 mm (SD: 17.8) with DC161‐DP0292 and −3.2 mm (SD: 20.0) with the comparator.

#### Second product application: T4–T6h

Following the second product application (administered at T4h), the adjusted mean difference in AUC (T4‐T6h) between DC161‐DP0292 and comparator for dryness of mouth was −382.69 (SD: 390.86; 95% CI: −1163.06, 397.69). The first postdose time point of assessment was scheduled 30 min after the second product application and 15 min after a meal. The mean change in VAS score for dryness of mouth was −20.5 mm (SD: 17.8) for DC161‐DP0292 and −18.4 mm (SD: 24.8) for the comparator.

Despite the different administration conditions for the first and second product applications, the magnitude of the effect observed 30 min after each application was similar.

#### Time to onset and duration of action

**Figure 2:**
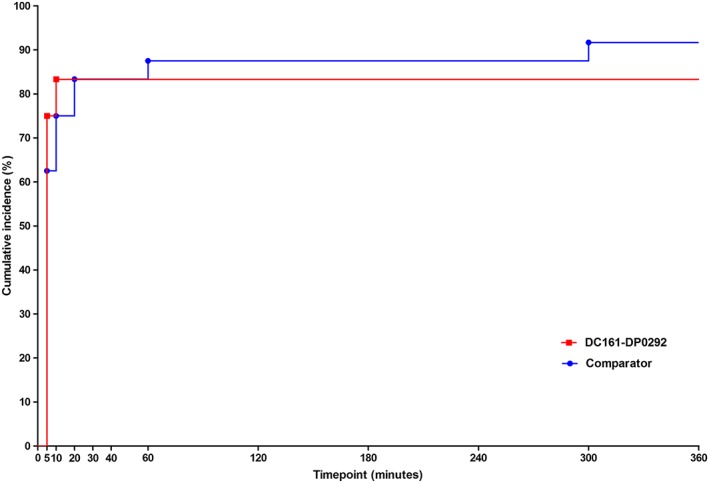
Cumulative incidence of time to onset of action (reduction of at least 20%): rate of dryness of mouth (visual analog scale item 4) (full analysis set).

The overall cumulative incidence of clinically significant action (defined as a reduction from baseline of at least 20% of dryness of mouth) was comparable for DC161‐DP0292 and the comparator (Fig. 2). However, time to onset of effect appeared to be shorter for DC161‐DP0292: 18 subjects (75%) had at least a 20% reduction from baseline at 5 min and 20 subjects (83.3%) at 10 min after the first application of DC161‐DP0292 versus 15 subjects (62.5%) and 18 subjects (75%) at 5 and 10 min, respectively, after application of the comparator (Fig. 2). The mean duration of action was 1 h 29 min (SD: 1 h 18 min) for DC161‐DP0292 and 1 h 08 min (SD: 1 h 29 min) for the comparator.

#### Other secondary efficacy results

Regarding the secondary criteria related to the other seven dry mouth symptoms, although these data were also highly variable, both formulations showed similar efficacy profiles to the primary analysis and supported the primary results. From T0 to T4h, DC161‐DP0292 seemed to reduce the intensity of the associated symptoms of xerostomia; although all CIs cross zero, a consistently greater mean improvement versus the comparator was observed, especially over the 4 h after the first product application (Table 4) and particularly during the first 90 min (data not shown). From T4h toT6h, after the second application, these items showed a similar time course of effect for DC161‐DP0292 and the comparator and were generally in accordance with the overall trend observed with item 4 (dryness of mouth). This suggested an improvement in all investigated dry mouth symptoms when the products were used prior to meal intake.

**Table 4:** Secondary efficacy analysis: VAS items 1, 2, 3, 5, 6, 7, and 8 of VAS – adjusted AUC difference for DC161‐DP0292 versus comparator (FAS).

VAS item	Adjusted AUC difference between DC161‐DP0292 and comparator (Aequasyal®)	
	T0–T4h	T4h–T6h
1 = difficulty in speaking		
LSM (SE)	−791.94 (525.11)	−124.83 (352.88)
[LSM 95%CI]	[−1840.36; 256.48]	[−829.38; 579.71]
2 = difficulty in swallowing		
LSM (SE)	−579.02 (620.40)	0.58 (385.49)
[LSM 95%CI]	[−1817.70; 659.65]	[−769.07; 770.24]
3 = saliva in mouth		
LSM (SE)	−683.17 (594.59)	−128.25 (365.16)
[LSM 95%CI]	[−1870.30; 503.97]	[−857.31; 600.81]
5 = dryness of throat		
LSM (SE)	−733.62 (587.84)	−368.02 (364.85)
[LSM 95%CI]	[−1907.28; 440.03]	[−1096.47; 360.43]
6 = dryness of lips		
LSM (SE)	−629.71 (759.60)	−23.79 (457.15)
[LSM 95%CI]	[−2146.30; 886.88]	[−936.53; 888.94]
7 = dryness of tongue		
LSM (SE)	−525.87 (548.39)	−165.60 (380.42)
[LSM 95%CI]	[−1620.77; 569.02]	[−925.13; 593.92]
8 = level of thirst		
LSM (SE)	−437.50 (704.91)	−49.60 (437.90)
[LSM 95%CI]	[−1844.90; 969.90]	[−923.90; 824.69]

VAS, visual analog scale; AUC, area under the curve; FAS, full analysis set; LSM, least square mean; SE, standard error; CI, confidence interval.

### Safety and tolerability

All 24 subjects received the four products and were included in the safety set. No clinically relevant abnormalities were detected with any product following systemic or local examinations. No serious adverse events or events leading to study product discontinuation or premature withdrawal were reported during the study.

Related treatment‐emergent adverse events were mainly reported in the gastrointestinal disorders system organ class (one event each after application of the DC161‐DP0292 formulation and comparator). There were no relevant differences between products in terms of incidence and type of related treatment‐emergent adverse event. Objective assessment of the oral mucosa showed no relevant safety signals, with mainly mild or moderate dryness of mouth tissue reported for most subjects (as would be expected in this subject population), with only one case of mild redness with DC161‐DP0292 but no signs of inflammation.

In all groups, local tolerability was assessed as very good (score of 0 for painful tongue, burning sensation, and tickling) by over 90% of subjects throughout the study following both product applications.

**Figure 3:**
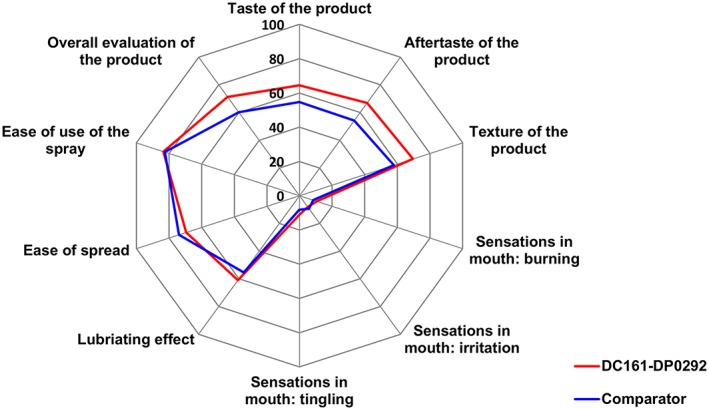
Product acceptability after the first application (full analysis set).

**Figure 4:**
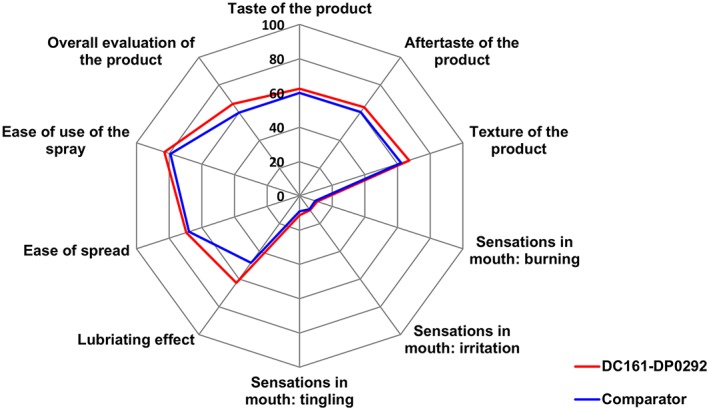
Product acceptability after the second application (full analysis set).

Product acceptability for the DC161‐DP0292 formulation and the comparator, as rated by subjects after the first and second product applications, is shown in Figures 3 and 4, respectively. Subjects' overall evaluation of the product was higher for DC161‐DP0292 than comparator following both applications. After the first application, acceptability of product taste, aftertaste, texture, and lubricating effect was higher for DC161‐DP0292. Sensations of burning, irritation, and tingling as well as ease of use of the spray were rated similarly for the two products following the first application, and only ease of spread was rated higher for the comparator (Fig. 3). After the second application, product acceptability for DC161‐DP0292 was higher or similar to the comparator for all rated items (Fig. 4).

## Discussion

Drug‐induced xerostomia is a common side effect associated with many pharmacological drug classes (Gupta *et al*., 2006). If the dry mouth condition becomes chronic, it may lead to important oral pathological changes, with increased risk of infections and caries (Porter *et al*., 2004). In addition, dry mouth can have a marked negative impact on the patient's quality of life as a result of the impairment of oral functions. Age and polypharmacy play a very important role in patients with xerostomia (Porter *et al*., 2004; Thelin *et al*., 2008). Consequently, in elderly patients, particularly those over 65 years of age, saliva substitutes represent the best palliative management option, although no ideal artificial saliva has been developed to date. Existing products replace the components and functions inherent to natural saliva, which are impaired as a result of dry mouth. Because of their lack of undesirable effects, saliva substitutes can be used for prolonged time periods as palliative or coadjuvant treatment, depending on the severity of hyposialia. Likewise, these products can be used as often as required and are adaptable to the needs of the individual patient and degree of oral dryness (Silvestre *et al*., 2009).

In the current study, three formulations (two aqueous and one oily) of DC161 oral spray, a new saliva substitute, and an active comparator, Aequasyal® oral spray (oily), were assessed for clinical efficacy in terms of their relief on drug‐induced xerostomia and other associated symptoms. The subjective intensity of various symptoms was self‐assessed by the subject using a 100 mm VAS, a validated and well‐described assessment tool.

Taking into account the overall variability of the data, as well as the sample size, only slight differences between the products could be deduced from the primary criterion, VAS item No. 04 “Dryness of your mouth”. None of the three DC161 formulations showed a statistically significant difference in effect versus the comparator. However, only the DC161‐DP0292 aqueous formulation (Elgydium Clinic Dry Mouth) showed a reduction in intensity of dryness of mouth versus the comparator (−935.00 [SE: 577.70; 95% CI: −2088.42, 218.42]) and was therefore selected for overall comparison. Although there were no statistically significant differences between the two products, the maximum effect on dryness of mouth following the first product application was marginally higher for DC161‐DP0292 than the comparator and occurred 5 min earlier. The onset of action, defined as a clinically relevant mean reduction of 20% from baseline, was observed within the first 5 min in 75% of subjects for DC161‐DP0292 (62.5% for the comparator).

Dry mouth is a sign as well as a symptom and is usually accompanied by generalized discomfort, eating and speaking disturbance (Eveson, 2008). As expected, the secondary criteria evaluated in this study showed an improvement in other symptoms, including swallowing and speaking, consistent with a reduction in mouth dryness. These effects were also observed when the spray was applied just before a meal (second product application).

Optimal topical treatment in xerostomia should provide fast relief and a long‐lasting effect, although published evidence in the literature is quite variable. For example, in one study (Silvestre *et al*., 2009), around 50% of patients showed immediate improvement with a new artificial saliva spray, and the average duration of effect was 15.3 min. For Mouly *et al*. (2007), improvement with saliva spray was only achieved from the second day for most patients, and average duration of effect was over 4 h. In our study, DC161‐DP0292 had a long duration of clinically relevant action (1 h 29 min ± 1 h 18 min), similar to that of the comparator (1 h 08 min ± 1 h 29 min).

The main study limitation was use of a subjective evaluation of dry mouth symptoms, which likely contributed to the large variability in responses observed between formulations. In addition, evaluations were performed after only two applications of each product and in a limited number of subjects, and these factors should therefore be taken into consideration. As the study was not blinded, both the investigator and subjects were aware of the formulations being investigated and could bias the subjective assessments. Also as the scheme of application differed between the test products and comparator, it was not blinded; however, subjects were randomized in four different sequence groups to test all four products, allowing comparison of all three DC161 formulations with the comparator. This study was designed as an exploratory study, and the analysis was not planned to be demonstrative (no rationale for sample size).

Topical treatment response in general depends directly on patient compliance, which can be jeopardized by poor local tolerability and acceptability. Discomfort sensations, as well as ease of use and flavor, are important factors to evaluate for the treatment of dry mouth with artificial saliva. In the study by Silvestre *et al*. (2009), flavor was rated as favorable by almost 50% of the test subjects. In the present study, besides very good local tolerability, the subject self‐rated assessment indicated that DC161‐DP0292 provided a slightly higher acceptability of taste/aftertaste, texture, and lubricating effect versus the comparator. In patients with xerostomia, because the properties of saliva are usually impaired, a saliva substitute not only maintains mouth humidity but also promotes a lubricating effect and therefore prevents the disorders related to impaired saliva secretion (Humphrey and Williamson, 2001).

## Conclusion

This study in 24 subjects with drug‐induced xerostomia was conducted to assess the efficacy, safety, and tolerability of three different new oral spray formulations of DC161 (two aqueous and one oily) and an active comparator Aequasyal® (oily). In general, analysis of the primary criterion showed that despite large variability among the products, the selected aqueous formulation – DC161‐DP0292 – could reduce the intensity of dryness of mouth at least as well as the comparator: DC161‐DP0292 provided fast relief and a long‐lasting effect on mouth dryness. Both DC161‐DP0292 and the comparator improved other symptoms associated with dry mouth such as swallowing and speaking, even when applied just before a meal. The DC161‐DP0292 formulation was well tolerated and rated by subjects as providing a slightly higher acceptability of taste/aftertaste, texture, and lubricating effect than the comparator. This new oral spray, presented as an aqueous formulation, DC1661‐DP0292, is therefore considered to be a relevant treatment for drug‐induced xerostomia, providing fast and long‐acting symptomatic relief while potentially improving patient compliance.

## Conflict of Interest

F. D. is an employee of SocraTec R&D, a company that received payment for study monitoring and medical writing. F. T., R. C., JPh. G., and J. B. are employees of Pierre Fabre Laboratories.

## Funding Information

Pierre Fabre Medical Devices provided funding for the study.
